# Frailty and Medication Appropriateness in Rural Adults: Proposing Interventions through Pharmacist–Physician Collaborative Efforts

**DOI:** 10.3390/jcm13195755

**Published:** 2024-09-27

**Authors:** Cristina García, José M. Ocaña, Mónica Alacreu, Lucrecia Moreno, Luis A. Martínez

**Affiliations:** 1 Community Pharmacy, 02161 Albacete, Spain; 2 Cátedra DeCo MICOF-CEU UCH, University Cardenal Herrera-CEU, 46115 Valencia, Spain; 3 Department of Pharmacy, University Cardenal Herrera-CEU, 46115 Valencia, Spain; 4 Servicio de Salud de Castilla-La Mancha (SESCAM), 02161 Albacete, Spain; 5 Department of Mathematics, Physics and Technological Sciences, University Cardenal Herrera-CEU, 46115 Valencia, Spain; 6 Department of Medical Sciences, School of Pharmacy, University of Castilla-La Mancha (UCLM), 02171 Albacete, Spain

**Keywords:** frailty, polymedication, potentially inappropriate medications, primary care, community pharmacy, rural

## Abstract

**Background:** Frailty and polymedication are closely interrelated. Addressing these concurrent conditions in primary care settings relies on the utilization of potentially inappropriate medication (PIM) lists and medication reviews (MRs), particularly in rural areas, where healthcare professionals serve as the sole point of access to the medical system. The aim of this study was to examine the relationship between medication appropriateness and variables related to frailty in a rural municipality in order to propose potential strategies for therapy optimization. **Methods**: This cross-sectional study included all adult community dwellers aged 50 and above officially registered in the municipality of Tiriez (Albacete, Spain) in 2023 (*n* = 241). The primary outcome variable was frailty (assessed using the fatigue, resistance, ambulation, illness, and loss of weight (FRAIL) scale). The independent variables were age, gender, medication regimen, history of falls, comorbidities, PIMs (evaluated using the screening tool of older persons’ prescriptions (STOPP) 2023 criteria), fall-risk-increasing drugs (FRID), and anticholinergic burden (ACB). **Results**: The prevalence of frailty was approximately 20%. FRID and ACB scores were statistically associated (*p*-value < 0.001) with frailty, 1.1 ± 1.3 vs. 2.5 ± 1.7, and 1.0 ± 1.3 vs. 2.8 ± 2.5, respectively. Regardless of age, frailty was observed to be more prevalent among females (odds ratio (OR) [95% confidence interval (CI)]: 3.5 [1.5, 9.0]). On average, 2.1 ± 1.6 STOPP criteria were fulfilled, with the prolonged use of anxiolytics and anti-peptic-ulcer agents being the most frequent. Priority interventions (PIs) included opioid dose reduction, benzodiazepine withdrawal, and the assessment of antidepressant and antiplatelet treatment plans. **Conclusions:** The optimization of medication in primary care is of paramount importance for frail patients. Interventional measures should focus on ensuring the correct dosage and combination of drugs for each therapeutic regimen.

## 1. Introduction

Frailty is a clinical syndrome characterized by a reduction in an individual’s capacity to cope with stressors and is a result of cumulative impairments in multiple physiological systems [1]. Polymedication typically refers to the concurrent use of five or more medications, although there is no consensus regarding this definition. The relationship between the two syndromes appears to be bidirectional [2].

From the perspective of the frail patient, appropriate treatment of comorbidities enables the effective management of chronic health problems, improves quality of life, minimizes the level of dependence, and increases life expectancy. Nevertheless, the term ‘polymedication’ is not without a certain negative connotation, as it is associated with the unnecessary and/or off-label use of drugs. In light of these considerations, the purely numerical criterion for categorizing a patient as polymedicated may prove to be of limited utility. When frailty represents the primary concern, the use of multiple medications should not automatically be regarded as a cause for concern, provided that they are appropriate [3].

Thus, managing medication appropriateness according to the clinical condition and desired clinical outcomes of a frail patient represents a pivotal aspect of his/her assessment. Additionally, when regularly monitoring frail adults, healthcare providers should consider deprescribing strategies to reduce the risk of adverse drug events and improve medication safety, and they should adjust medication regimens when needed. Moreover, the role of variables such as age and gender in any potential intervention must be considered given their well-documented correlation with frailty and the use of multiple medications.

A medication review (MR) is a systematic assessment of the medications taken by a patient to ensure safe and effective use and to achieve optimal health outcomes [4]. The MR is a core component of the comprehensive geriatric assessment, as it enables the identification of potentially inappropriate medications (PIMs). The lack of available evidence on the benefits of specific drugs in frail patients, together with the strong association between polymedication and frailty, makes deprescribing a very appropriate strategy [5].

Many tools are employed in routine clinical practice for the screening of frailty and the assessment of medication appropriateness. Some are particularly well suited for use by a primary care physician and/or a community pharmacist operating in rural areas, as they facilitate rapid screening without the need for specific instrumentation. In the context of deprescription, particular emphasis is placed on medications that may contribute to an elevated risk of falls and/or have a high anticholinergic burden [6]. The most widely used criteria for assessing PIMs worldwide are the Beers and the screening tool of older persons’ prescriptions (STOPP) criteria [7,8]. In the specific context of community pharmacy, tools such as pharmacotherapeutic follow-up and compliance aid systems, which are designed to identify drug-related problems and address the negative outcomes of medication (including non-adherence issues) [9], are particularly well suited for the management of frail patients.

Primary care services (general practice and community pharmacy) act as the ‘front door’ to the health system. In rural areas, these professionals serve as the unique point of access to healthcare for each individual and maintain close and continuous contact with all patients. This practice setting offers an optimal environment for the MR, the identification of PIM, the acquisition of valuable information for deprescribing, and the development of coordinated strategies based on the results obtained.

The objective of this study is to assess the relationship between medication appropriateness and variables related to frailty in a rural population and to propose potential strategies for optimizing medication in frail patients through tailored multidisciplinary interventions.

## 2. Materials and Methods

### 2.1. Participants and Study Procedure

We conducted a descriptive cross-sectional study including all dwellers officially listed as residing within the municipal boundaries (Tiriez, Spain. Population: 463 inhabitants) [10]. The inclusion criteria were as follows: adults aged 50 years and older, enrolled with the local primary care physician, receiving any chronic pharmacological treatment dispensed at the local community pharmacy in 2023, and willing and able to provide written informed consent. Individuals receiving only acute treatments (taken for less than 3 months) were excluded from the study.

### 2.2. Data Collection

Data were collected from the participants’ electronic medical and dispensing records. Information on demographic variables (age and gender), medication regimen (categorized according to the Anatomical Therapeutic Chemical classification system, level 5) [11], history of falls, and comorbidities was collected. A database containing all anonymized data was created. Data cleansing was carried out according to the inclusion and exclusion criteria, which resulted in a final sample of 241 individuals included in the study (see Appendix A).

Frailty was assessed using the fatigue, resistance, ambulation, illness, and loss of weight (FRAIL) scale [12], which is a concise instrument comprising five straightforward yes/no questions designed to evaluate the characteristics of the frailty phenotype, as proposed by Fried [13], and allows the time-efficient identification of individuals exhibiting these signs. The questionnaire assesses self-reported presence/absence/level of fatigue, resistance, ambulation ability, illnesses, and unintentional loss of weight (>5%). A value of 1 is assigned to each response, thus resulting in a frailty score that ranges from 0 to 5 points. A score of 3 or above is indicative of frailty, whereas a score of 1 or 2 is associated with a prefrailty status. Individuals scoring 0 are considered non-frail. The 2022 Spanish National Health System expert recommendations propose that individuals with a score above 0 on the scale be considered frail, with the objective of enhancing sensitivity in primary healthcare [14].

The use of drugs with anticholinergic activity was assessed using the STOPP criteria [8] (version 2023, section M: concomitant use of two or more drugs with antimuscarinic/anticholinergic properties) and was considered in a separate section (see Section 3). Subsequently, the anticholinergic burden (ACB) was quantified using the CRIDECO Anticholinergic Load Scale (CALS) [15], which was developed by our research group. It includes 217 drugs with anticholinergic activity, graded according to their potency (1—low; 2—medium; and 3—high). A total score greater than 3 is considered a clinically relevant ACB [16]. All drugs displaying an anticholinergic load were initially identified and then incorporated into the calculations, subjected to a comprehensive assessment, and considered potential candidates for subsequent therapeutic interventions.

Fall-risk-increasing drugs (FRID) were assessed using STOPP criteria (version 2023, section K: drug classes that predictably increase fall risk) and were considered in a separate section (see Section 3). For each patient, the number of FRIDs was calculated [17].

A comprehensive MR was conducted for all frail patients in accordance with Spanish national guidelines [9]. Briefly, the review entailed a patient interview, during which data regarding clinical and biological parameters, medication use, adherence, and health concerns were collected. Medication appropriateness was assessed in accordance with the STOPP criteria (2023 version, sections A–J, and L). In order to formulate potential interventions, the appropriateness of the medication was individually assessed for every frail patient and thoroughly discussed by the professionals involved in their care. A plan for intervention was developed, with the patient’s involvement also sought when necessary.

### 2.3. Statistical Analysis

A descriptive statistical analysis was carried out using R software (R-4.4.1, R Foundation for Statistical Computing, Vienna, Austria). The sample was analyzed using the average and standard deviation for quantitative variables. To ascertain whether there were any statistically significant differences between the means and medians of quantitative variables between the frail and non-frail patient groups, the Wilcoxon and Mann–Whitney tests were employed, respectively. Chi-square and Fisher’s exact tests were employed for the analysis of qualitative variables.

Multivariate logistic regression models were fitted to estimate the probability of frailty. The goodness-of-fit of the regression models was analyzed with the Hosmer–Lemeshow test. Statistical significance was indicated by * if the *p*-value of the test < 0.05, ** if the *p*-value < 0.01, and *** if the *p*-value < 0.001.

### 2.4. Ethical Approval

The study was approved by the Research Ethics Committee at the Universidad CEU Cardenal Herrera (approval no. CEI22/249). In accordance with the Declaration of Helsinki, all of the participants gave their written informed consent to participate.

## 3. Results

The majority (52%, *n* = 241) of individuals registered within the municipality are patients over the age of 50 years with long-term medication regimens. Table 1 provides an overview of the variables examined in relation to patient frailty. A total of 20.8% of the patients exhibited a score indicative of frailty. The prevalence rose to 40% when the age was limited to 65 and above. Age and gender were significantly associated with frailty, with the condition being more commonly observed among females, as well as in individuals aged 80 and above. The number of medications (NOM) was twofold higher among frail patients compared to their non-frail counterparts (10 vs. 5). The use of pharmaceutical agents with documented associations with falls and the anticholinergic load was also significantly associated with frailty.

**Figure 1:**
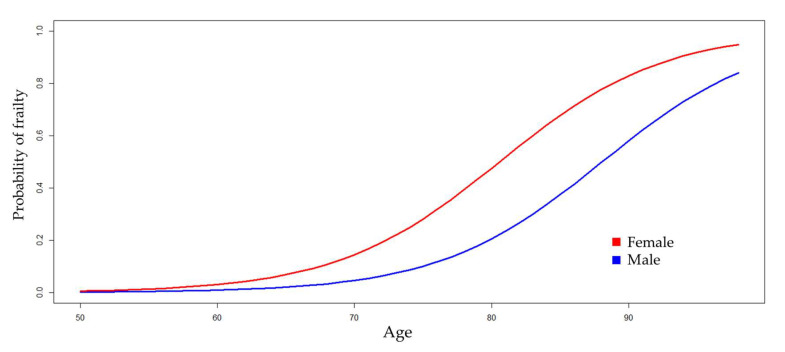
Model 1: estimation of the probability of frailty according to the age and gender of the patients.

Table 2 shows four logistic regression models for estimating the probability of frailty. Model 1 was constructed by incorporating the non-modifiable variables (age and gender) of the patient. The results indicate that, in individuals of the same age, being female can increase the odds ratio (OR) of frailty by as much as nine times, with 95% confidence. Similarly, in individuals of the same gender, with each year of aging, the OR increases by 13% to 25% (Figure 1).

Three multivariate logistic regression models were fitted, alternatively incorporating one modifiable variable into Model 1: Model 2 incorporates the NOM, Model 3 FRIDs, and Model 4 the CALS score. The ORs for gender and age show a notable degree of stability across all models. Model 4 suggests that the anticholinergic load has a greater impact on frailty than the NOM or FRIDs. For individuals of the same gender and age, each additional anticholinergic unit increases the OR by a factor of 1.2 to 2. An additional model was constructed using the five variables under investigation. In this model, the statistical significance of the NOM and FRIDs was lost, while the anticholinergic load maintained its significance.

Table 3 shows (i) a summary of the PIMs detected during the MRs performed for frail patients and (ii) the proposed intervention strategies designed and coordinated between the primary care physician and community pharmacist. Recommendations were classified as priority interventions (PIs) if their implementation was feasible within our context and with the resources at our disposal. Conversely, those that necessitated referral to other levels of care or specialized follow-up were designated as complex interventions (CIs).

Anticholinergic drugs were prescribed to 94% of the patients under review, with a mean CALS score of 3 (ranging from 1 to 10). The most potent agents were urinary antispasmodics, while the primary contributors to the overall load were found to be antidepressants. A total of 46 patients were taking at least one FRID, with an average of 2.3 ± 1.6 per patient, ranging from 1 to 7. Antidepressants and diuretics were the FRIDs most frequently identified as PIMs. An average of 2.1 ± 1.6 STOPP criteria (range 0–6) were fulfilled. Two patients did not fulfill any STOPP criteria. Treatment with anxiolytics for a period exceeding 4 weeks and the prolonged use (longer than 8 weeks) of peptic ulcer and gastroesophageal reflux agents were the most frequently recorded.

## 4. Discussion

There is an intimate relationship between frailty and aging. This issue is of particular concern in rural environments, where the rates of aging and population decline are even more pronounced than in urban contexts. However, the prevalence of frailty in rural areas has been explored in a limited number of studies [18]. Those conducted in Spain have reported a prevalence of frailty ranging from 8.4% to 20.4% [19,20,21,22]. The frailty and dependency in Albacete (FRADEA) study, which was conducted in our geographical area, reported a frailty prevalence of 65.4% among individuals over the age of 70 [23]. Our data revealed a prevalence of 45.2% in this age group. The observed differences may be attributed, to some extent, to the distinctive characteristics of the populations (the FRADEA study included institutionalized patients). Further investigation is warranted to ascertain the influence of rurality on frailty and health outcomes.

All multivariate regression models revealed that the OR of developing frailty is heightened in females (see Table 2). The relationship between the female sex and frailty prevalence has been a subject of long-standing recognition and is thus termed the ‘sex–frailty paradox’ [24]. In addition, the incidence of frailty and prefrailty was also higher in women than in men [25]. Moreover, given the holistic nature of frailty, it is plausible that psychosocial variables or behaviors exert an influence on the syndrome. Since personal and medical factors contributing to social vulnerability and frailty are related, women may be more socially vulnerable than men, which could, in turn, contribute to their higher frailty levels [26]. It can be argued that PIM criteria and deprescribing tools that include gender as a factor in determining frailty may be lacking [27].

Regular assessments of pharmacological treatments at crucial stages are essential to guarantee that frail individuals continue to receive secure and appropriate medications. The involvement of a primary care physician and a pharmacist in the MR process has been demonstrated to positively impact the implementation of recommendations [28,29]. Furthermore, bidirectional communication between healthcare professionals has been shown to reduce the prevalence of duplicate treatments, which represents the most frequently cited STOPP criterion [30].

A substantial body of literature exists on the use of tools to detect PIMs in Spain or Europe [31,32]. The most frequently reported PIMs in community-dwelling patients were benzodiazepines, antiplatelet agents, proton pump inhibitors, and opioids. To the best of our knowledge, no studies have been conducted that utilize the most recent update of the STOPP criteria. However, our data largely align with the findings reported in the existing literature, and the appropriateness of medication use in our frail patient sample is satisfactory overall.

The aforementioned tools fail to account for individual patient information, including psychosocial or/and behavioral variables, which could prove relevant. A crucial element of deprescribing is the active involvement of patients, who must assume a primary role in the management of and compliance with their treatment plans. Recurrent face-to-face interactions inherently entail the acquisition of information regarding psychosocial and personal contexts. Given the prevailing structure of Spanish Healthcare Systems [33], overcoming this limitation in rural settings seems more feasible than in urban contexts.

The assessment of frailty in rural primary care settings represents a significant challenge that necessitates a personalized approach. In this context, the STOPP criteria should be regarded as a preliminary screening tool, the results of which can be used to define an initial list of priorities. Using this approach facilitates deprescription in frail patients in real-world settings [34]. Regardless of the existence of deprescription criteria, and since the process may be complex, the risk–benefit ratio of any given intervention should be thoroughly evaluated. In fact, clinical practice guidelines already include frailty as a therapeutic determinant [27]. When reviewing the medication of a frail patient, it is essential to keep all of this in mind. A collaborative analysis of the collected data allows for the development of tailored interventions that should be discussed from the perspective of frailty.

Four out of five frail patients were treated with an anti-peptic-ulcer agent. The rationale for this is that proton pump inhibitors are indicated as prophylactics in prolonged treatments with acetylsalicylic acid in Spain [35]. A review of the medical record and an assessment of the necessity for an antiplatelet agent are proposed to be the primary focus of the intervention. If needed, gradual withdrawal is advised to avoid rebound acid hypersecretion.

Metformin is the treatment of choice for the management of diabetes mellitus in frail patients [36]. Despite its anticholinergic activity, treatments with metformin are considered appropriate, except in cases of chronic kidney disease (stage 4).

The inappropriate use of acetyl-salicylic acid in the primary prevention of cardiovascular diseases can be easily assessed. Moreover, deprescription of the antiplatelet agent eliminates the need for gastroprotection. A more detailed analysis is required for treatments with anticoagulants. It is advisable that patients undergoing concurrent treatment with both direct oral anticoagulants (DOACs) and glycoprotein P inhibitors be monitored regularly for bleeding risk assessment. As far as anti-vitamin K appropriateness is concerned, switching to DOAC therapy in frail patients has been associated with an increased risk of bleeding without a reduction in thromboembolic events [37]. Thus, any change should be carefully considered.

Furosemide is used in conjunction with other drugs to manage hypertension. When modifying a furosemide-based treatment plan, several factors must be considered, including the presence of kidney disease, edema, inadequate blood pressure control, or other comorbidities. In most cases, a gradual dose adjustment may prove more appropriate than treatment withdrawal [38].

The decision to utilize statin therapy in frail patients is contingent upon their functional status and age. In patients under 75 years of age, treatment is recommended in accordance with the level of cardiovascular risk. For patients over 75, a comprehensive geriatric assessment is recommended, given that their functional status exerts as much influence as traditional cardiovascular factors on the risk of mortality [39]. Statins for primary cardiovascular prevention in persons aged 85 and over with established frailty is a STOPP criterion [8].

The use of chronic opioids may increase the risk of prolonged sedation or confusion. Additionally, due to their ACB, they may increase the risk of falls and subsequent fractures [40]. It is preferable to reduce the dosage using pharmacological alternatives without anticholinergic activity.

Benzodiazepines and their analogs are clear candidates for deprescription in frail patients. This is a complex and time-consuming process that is particularly challenging to implement in a primary care setting, thus necessitating a referral. Several programs involving different professionals have been proposed for deprescription, and they have shown that a gradual reduction in the drug dosage may be an effective strategy. Furthermore, it is crucial to coordinate and agree on decisions with the patient throughout the process. Cognitive behavioral therapy is a useful element in this process [41].

Among the frail patients assessed, approximately 60% were treated with antidepressants. Frailty and depression share symptomatology and risk factors. Moreover, frail patients may not respond to antidepressant medication as well as their non-frail counterparts [42]. Primary care professionals can easily assess the control of the pathology through validated tests [43]. Further interventions require a specialist physician referral.

Urinary antispasmodics are the drugs with the greatest anticholinergic load. Depending on the degree of symptom control, dose tapering or deprescription can be attempted. Mirabegron may be a non-anticholinergic alternative treatment option. Non-pharmacological treatments represent an additional effective therapy, although they necessitate the involvement of qualified professionals [44,45].

Some limitations must be mentioned: (i) This study was conducted in a single municipality. This, in turn, enables an exhaustive examination of the population and facilitates the tailoring of effective interventions. While we do not intend to generalize, a multicenter study may yield more valuable insights. (ii) The design is cross-sectional, which precludes the possibility of establishing a causal relationship between the appropriateness of the medication and any variable or documented health outcome. This goal falls within the scope of a broader, more ambitious investigation. Given the complexity of the task at hand and the necessity of interdisciplinary collaboration, we believe that it is not viable to pursue this undertaking as a standalone investigation.

## 5. Conclusions

Further research is required to ascertain the impact of gender on the phenomenon of frailty. The development of appropriate tools for the assessment of PIMs and the process of deprescribing with gender included as a determinant may prove to be an effective means of advancing this field of study.

Interventional measures in primary care are of paramount importance for frail patients. It is essential that tailored approaches be designed to ensure that each patient is treated with the drugs required for their specific therapy within the appropriate risk–benefit parameters. Thus, the most effective intervention in medication is individual optimization.

## Figures and Tables

**Table 1:** Descriptive statistical analysis of the variables associated with frailty.

Variables	Groups	Total n (%) 241 (100)	Non-Frail *n* (%) 191 (79.3)	Frail *n* (%) 50 (20.8)	*p*-Value
Gender	Male	106 (44.0)	93 (48.7)	13 (26.0)	0.004 a **
	Female	135 (56.0)	98 (51.3)	37 (74.0)	
Age		68.9 ± 12.8	64.9 ± 10.8	83.9 ± 7.7	<0.001 b ***
		66.0 [50.0, 98.0]	62.0 [50.0, 94.0]	85.0 [57.0, 98.0]	<0.001 c ***
	[50, 65)	116 (48.1)	115 (60.2)	1 (2.0)	<0.001 d ***
	[65, 80)	62 (25.7)	52 (27.2)	10 (20.0)	
	[80, 98]	63 (26.1)	24 (12.6)	39 (78.0)	
NOM		6.2 ± 4.4	5.2 ± 3.7	10.0 ± 5	<0.001 b ***
		5.0 [1.0, 21.0]	4.0 [1.0, 17.0]	10.0 [3.0, 21.0]	<0.001 c ***
FRID		1.4 ± 1.5	1.1 ± 1.3	2.5 ± 1.7	<0.001 b ***
		1.0 [0.0, 7.0]	1.0 [0.0, 6.0]	2.0 [0.0, 7.0]	<0.001 c ***
Benzodiazepines and z-drugs		62 (25.7)	42 (22.0)	20 (40.0)	0.009 a **
Antidepressants		63 (26.1)	33 (17.3)	30 (60.0)	<0.001 a ***
Opioids		28 (11.6)	15 (7.9)	13 (26.0)	<0.001 a ***
Diuretics		81 (33.6)	54 (28.3)	27 (54.0)	<0.001 a ***
CALS score		1.3 ± 1.8	1.0 ± 1.3	2.8 ± 2.5	<0.001 b ***
		1.0 [0.0, 10.0]	1.0 [0.0, 7.0]	2.0 [0.0, 10.0]	<0.001 c ***
	CALS score < 3	199 (82.6)	167 (87.4)	32 (64.0)	<0.001 a ***
	CALS score ≥ 3	42 (17.4)	24 (12.6)	18 (36.0)	
Treated with	<2 drugs with ACB	173 (71.8)	150 (78.5)	23 (46.0)	<0.001 a ***
	≥2 drugs with ACB	68 (28.2)	41 (21.5)	27 (54.0)	

NOM: number of medication; FRID: fall-risk-increasing drug; CALS: CRIDECO Anticholinergic Load Scale; ACB: anticholinergic burden. Frequency (%) is reported for qualitative variables, and mean ± standard deviation and 50th percentile [minimum, maximum] are reported for quantitative variables; a: Chi-square test; b: Wilcoxon test; c: Mann–Whitney test; d: Fisher’s exact test; **: *p*-value < 0.01; and ***: *p*-value < 0.001.

**Table 2:** Logistic regression models to estimate the probability of frailty.

		Model 1: Gender + Age	Model 2: Gender + Age + NOM	Model 3: Gender + Age + FRID	Model 4: Gender + Age + CALS
Variables	Groups	OR [95% CI]	OR [95% CI]	OR [95% CI]	OR [95% CI]
Gender	Male	1	1	1	1
	Female	3.5 [1.5, 9.0] **	3.6 [1.5, 9.6] **	3.2 [1.3, 8.4] *	3.4 [1.3, 9.3] *
Age		1.2 [1.1, 1.2] ***	1.2 [1.1, 1.2] ***	1.2 [1.1, 1.2] ***	1.2 [1.1, 1.2] ***
NOM		-	1.1 [1.0, 1.3] **	-	-
FRID		-	-	1.4 [1.1, 1.9] *	-
CALS score		-	-	-	1.5 [1.2, 2.0] **
*p*-value of the Hosmer–Lemeshow test		0.5290	0.8413	0.6428	0.7969
Akaike Information Criterion		146.0	140.1	141.1	134.5

NOM: number of medication; FRID: fall-risk-increasing drug; CALS: CRIDECO Anticholinergic Load Scale; OR: odds ratio; 95% CI: 95% confidence interval for the OR; *: *p*-value < 0.05, **: *p*-value < 0.01; and ***: *p*-value < 0.001.

**Table 3:** The review of potentially inappropriate medication (PIMs) and proposed intervention strategies in frail patients.

**Drug**	**ACB**	**FRID**	**STOPP Criteria (*n*)**	**Primary Care Interventions**	**Type**
A02B—Peptic Ulcer Agents					
Omeprazole	0	no	A1: Prescribed without a clinical indication (28). F2: Proton pump inhibitor for uncomplicated peptic ulcer disease at full therapeutic dosage for > 8 weeks (2).	Comprehensive review of the medical and pharmaceutical records. Withdrawal of the drug in the absence of an appropriate indication.	PI
Pantoprazole	0	no			
Rabeprazole	0	no			
Lansoprazole	0	no			
Esomeprazole	0	no			
A10B—Hypoglycemic Drugs					
Metformin	1	no	-	Drug of choice in frailty.	‡
B01A—Antithrombotic Agents					
Acetylsalicylic acid	0	no	C16: Acetylsalicylic acid for primary prevention in cardiovascular disease (11).	Comprehensive review of the medical and pharmaceutical records to confirm the history of cardiovascular events. Reconsider treatment appropriateness.	PI
Direct oral anticoagulants (DOAC)	0	no	C14: DOAC and P-glycoprotein drug efflux pump inhibitors (12).	Assess the risk of bleeding. Evaluate P-glycoprotein inhibitor deprescription.	CI
Anti vitamin K (Acenocoumarol)	0	no	C11: Vitamin K antagonist as a first-line anticoagulant for atrial fibrillation (3).	Comprehensive review of the medical and pharmaceutical records. Reconsider treatment appropriateness.	CI
C03—Diuretics					
Furosemide	1	yes	B10: Loop diuretic for treatment of hypertension with concurrent urinary incontinence (9).	Monitor adverse reactions.	CI
Spironolactone	0	yes	B13: Aldosterone antagonists together with other drugs may increase potassium levels (2).	Monitor serum levels.	PI
C10—Lipid-Modifying Agents					
Statins	0	no	B16: Statins for primary cardiovascular prevention in persons aged ≥ 85 and established frailty (7).	Comprehensive review of the medical and pharmaceutical records to confirm the history of cardiovascular events.	PI
				Reconsider treatment appropriateness.	CI
N02—Analgesics					
Tramadol	2	yes	L1: Strong oral or transdermal opioids as first-line treatment for mild pain (11). L2: Use of daily opioids without concomitant laxatives (10).	Gradual reduction in the drug dosage and withdrawal. Alternative: paracetamol or NSAIDs. Add laxative.	PI
Tapentadol	1	yes			
Fentanyl	1	yes			
Buprenorphine	0	yes			
Gabapentin, pregabalin	0	no	L5: Gabapentinoids for non-neuropathic pain (4).	Evaluate pain type. Alternative: paracetamol or NSAIDs.	PI
N05—Psycholeptics					
Lorazepam	1	yes	D8: Benzodiazepines ≥ 4 weeks (21).	Referral and gradual withdrawal. Cognitive behavioral therapy. Evaluate the use of melatonin in patients receiving benzodiazepine for insomnia.	CI
Diazepam	1	yes	G4: Benzodiazepines with acute or chronic respiratory failure (4).		
Potassium chlorazepate	1	yes	A3: Duplicate drug class prescription (diazepam-potassium chlorazepate) (1).		
Zolpidem	0	yes	D11: Z-drugs for insomnia ≥ 2 weeks (1).		
N06A—Antidepressants					
Amitriptyline	3	yes	D2: Initiation of TriCyclic Antidepressants as first-line treatment for major depression (1).	Assess the origin of the symptomatology.	PI
			A3: Duplicate drug class prescription (amitriptyline-duloxetine) (1).		
Paroxetine	2	yes			
Sertraline	1	yes	A3: Duplicate drug class prescription (sertraline-mirtazapine) (1).		
Trazodone	1	yes			
Duloxetine	0	yes	I7: Duloxetine in the presence of urinary urgency or urinary incontinence due to urgency (1).	A specialist physician referral is suggested for deprescription. Prioritize drugs without anticholinergic load.	CI
Escitalopram	1	yes			
Mirtazapine	1	yes			
Fluoxetine	1	yes			
Desvenlafaxine	1	yes	A3: Duplicate drug class prescription (desvenlafaxine-duloxetine) (1).		
G04BD—Urinary Antispasmodics					
Fesoterodine	3	yes	I1: Systemic antimuscarinic drugs in the presence of dementia or chronic cognitive impairment (1).	Assess the effectiveness of the antispasmodic. Alternative: mirabegron. Non-pharmacological treatments are suggested.	PI
Solifenacine		yes			

ACB: anticholinergic burden; FRID: fall-risk-increasing drug; STOPP: screening tool of older persons’ prescriptions; NSAIDs: non-steroidal anti-inflammatory drugs PI: priority intervention; CI: complex intervention; ‡ no intervention (see Discussion).

**Figure A1:**
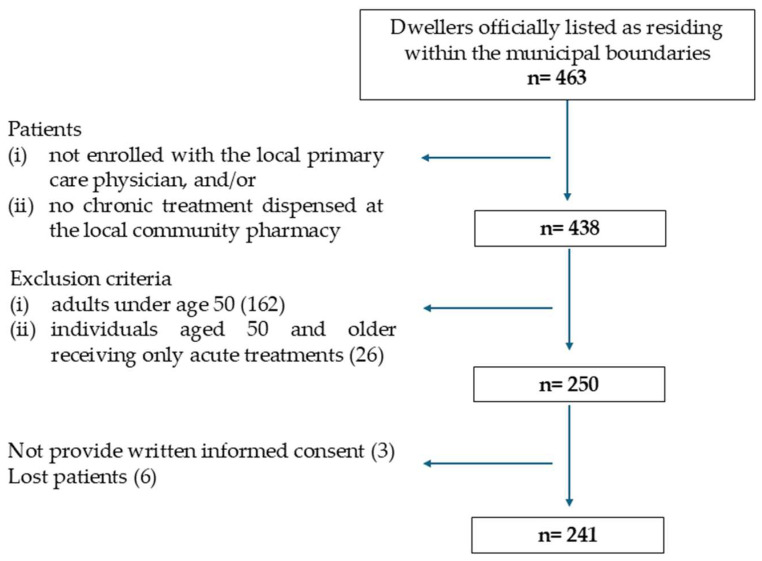
Flow chart of the population screening process.

## Data Availability

Data are available upon request.
